# Effect of Long-Term Isatin Administration on Daily Physical Activity and Cardiac Performance in Female Rats

**DOI:** 10.5152/eurasianjmed.2025.25763

**Published:** 2025-10-30

**Authors:** Selma Arzu Vardar, Muhammed Ali Aydın, Orkide Palabıyık, Ecem Büşra Değer, Esra Akbaş, Nihayet Fırat, Selen Yıldız, Necdet Süt

**Affiliations:** 1 Department of Physiology, Trakya University Faculty of Medicine, Edirne, Türkiye; 2 Department of Medical Services and Techniques, Trakya University Health Services Vocational College, Edirne, Türkiye; 3 Department of Physiology, Namık Kemal University Faculty of Medicine, Tekirdağ, Türkiye; 4 Trakya University Institute of Health Sciences, Edirne, Türkiye; 5 Department of Audiology, Sakarya University of Applied Sciences Faculty of Health Sciences, Sakarya, Türkiye; 6 Department of Biostatistics, Trakya University Faculty of Medicine, Edirne, Türkiye

**Keywords:** Isatin, cardiac hypertrophy, extracellular-regulated kinase 1, 2, natriuretic peptide, voluntary running

## Abstract

**Background:**

Isatin, an endogenous indole found in the brain and peripheral tissues, has a wide spectrum of physiological and pharmacological effects. This study aims to disclose the impact of long-term isatin administration on daily voluntary running, cardiac performance, and the expression of genes and proteins involved in signaling pathways in left ventricular tissue in rats.

**Methods:**

Wistar Albino rats were housed in standard cages or cages with running wheels for 28 days and received either intraperitoneally saline or isatin at 20 mg/kg/day or isatin 100 mg/kg/day from day 14 until 28. The hearts were perfused with Krebs-Henseleit solution ex vivo to measure developed left ventricular pressure and rate of contraction and relaxation. Protein kinase B (AKT), extracellular signal-regulated kinase1/2 (ERK1/2), and pyruvate dehydrogenase kinase-4 (PDK4) gene and protein expressions were determined in the ventricle.

**Results:**

Isatin did not alter daily running activity, cardiac performance, or AKT gene expression in groups (*P *> .05 for all). Ventricular weight/body weight and ERK1/2 gene expression were higher in the physically active group administered a high dose of isatin (100 mg/kg/day) than in the inactive group administered the same dose (*P *= .007, *P *= .042, respectively). PDK-4 protein level was lower in the physically active group administered a low dose of isatin compared with the inactive control group.

**Conclusion:**

Long-term isatin administration is well tolerated in female rats without negatively affecting daily physical activity and ex vivo cardiac performance. In physically active rats, the ERK1/2- and PDK-4-mediated effects of isatin on the left ventricle may differ depending on its dose.

**Main Points**

- Isatin is an endogenous indole in the brain, heart, and peripheral tissues. It is also a pharmacological agent reported to cause acute immobility in rodents. The findings of this study showed that long-term administration of isatin at low or high doses does not decrease the level of voluntary daily physical activity in female rats.
- Cardiac hemodynamic performance does not differ in ex vivo-perfused rat hearts after administering high or low doses of isatin under physically active or sedentary conditions.
- Differences in molecular factors and ventricular growth were observed with high and low doses of isatin in physically active female rats. Isatin, administered in a high dose, may induce ERK-mediated ventricular growth, but low doses may suppress cyclic guanosine monophosphate (cGMP) and decrease the PDK-4 level in the left ventricle.

## Introduction

Isatin (1H-indole-2,3-dione) is an endogenous indole that binds natriuretic peptide and monoamine oxidase B receptors and, therefore, may act as a natriuretic peptide receptor blocker and cyclic guanosine monophosphate (cGMP) inhibitor.^1^^,^^2^ Despite promising data from anticancer, anticonvulsant, and antiviral clinical perspectives of isatin and its analogs, acute isatin administration has been shown to reduce animal mobility.^3^^-^^6^ Following the acute isatin injection, mice moved less^4^^,^^5^ and increased immobility was observed in rats receiving a single dose of isatin.^3^^,^^6^ However, information on the long-term treatment of isatin on daily physical activity (PA) is scarce. Therefore, the authors investigated the effect of long-term isatin administration on voluntary running activity in rats.

Studies in rodent models suggest that voluntary PA may differ depending on sex as a unique biological variable in rodent behavior.^7^ Female rats are more willing to perform voluntary PA than male rats.^8^ For this reason, the authors preferred female rats in this study, where the effects of voluntary PA are expected to be evident.

With respect to the cardiovascular system, isatin treatment decreases blood pressure,^9^ most likely due to its vasodilator effect on smooth muscles.^10^ Moreover, cardio-depressive effects have been observed in isolated frog hearts.^9^ However, the effect of isatin on cardiac performance and contractility is unclear. The authors’ previous study observed that ex vivo-perfused hearts obtained from rats injected with a single dose of isatin did not show a negative effect on cardiac contractility.^11^ Therefore, the authors examined the effect of long-term isatin administration in combination with prolonged increased daily PA on cardiac growth and a number of signaling pathways and cGMP release in the isolated perfused heart.

The effects of different doses of isatin on PA and cardiac tissue are unknown. A recent study reported that locomotor activity in mice was not affected by the application of isatin at a low dose of 15-20 mg/kg.^5^ However, ameliorative effects were reported on motor activity in the Parkinson’s model when isatin was administered at 100 mg/kg.^12^ Therefore, the present study aimed to determine the effects of different doses of isatin on voluntary PA in rats using relatively low and high isatin concentrations.

In this study, left ventricular expression of protein kinase B (AKT) and extracellular signal-regulated kinase1/2 (ERK1/2) levels were measured. As a molecular signal pathway marker, increased AKT activation prevents the loss of contractile cells.^13^ Extracellular signal-regulated kinase1/2 increases cardiac hypertrophy and function.^14^ Moreover, upregulation of pyruvate dehydrogenase kinase-4 (PDK4) impairs ventricular function,^15^ whereas its inhibition improves diastolic function.^16^ Therefore, the authors explore alterations of AKT, ERK1/2, and PDK-4 and B-type natriuretic peptide (BNP) as a natriuretic peptide associated with cardiac function^17^ both at the gene and/or protein level, in the left ventricle (LV) following isatin administrations in rats under conditions with or without voluntary PA.

## Materials and Methods

### Animals

Eighty-four Wistar Albino female rats (200-280 g) were obtained from the Experimental Animals Unit of Trakya University. All rats were housed in cages individually at a controlled temperature of 22 ± 1°C and humidity of 50-60% with a 12-hour dark–light cycle. During the experimental period, all animals in groups PA and C were kept in the same room and under the same environmental conditions. The rats in the groups had free *ad libitum* access to purified water and were fed with standard laboratory chow (Optima Rat Chow-Bolu, Türkiye). The experimental protocol was approved by the Trakya University Animal Experiments Local Ethics Committee (Approval No: TU-HADYEK-2021.02.01, Date: 26.02.2021). This study was carried out with the recommendations in the Guide for the ARRIVE 2.0.

**Figure 1.**
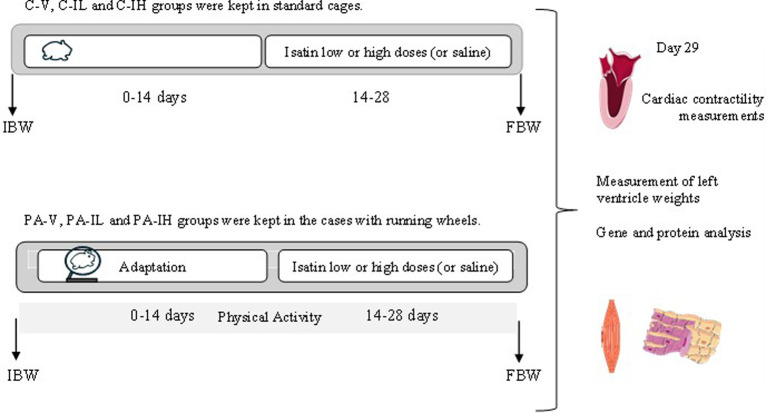
Study design. IBW, Initial body weight; FBW, Final body weight; C-V, control-vehicle; C-IL, control-isatin-low-dose; and C-IH, control-isatin-high-dose. PA-V, physical activity-vehicle; PA-IL, physical activity-isatin-low-dose; PA-IH, physical activity-isatin-high-dose groups.

The animals were divided into the PA and control (C) groups. In the PA groups, the first 14 days allowed the rats to adapt to voluntary exercise. Rats performing more than 2000 running cycles/day on at least 1 day in the last week of this adaptation period were included in the study. These rats were divided into physical activity-vehicle (PA-V), physical activity-isatin-low-dose (PA-IL), and physical activity-isatin-high-dose (PA-IH) groups, and control rats were divided into C-IL (daily injection with low dose of isatin), C-IH (daily injection with high dose of isatin), and C-V (daily injection with saline) groups (Figure 1). Each group consists of 14 animals and is divided into 2 subgroups.

In subgroup 1, the heart was mounted on a Langendorff-perfusion apparatus; in subgroup 2, heart weight and left ventricular weight were measured, and aliquots of left ventricular tissue were used for RNA and protein analysis. The body weights were measured before and after the experimental period (Figure 1).

The animal model used in this study was rats since they are widely used in animal studies on the effects of different doses of isatin,^18^ and substantial concentrations of isatin were determined in rat tissues in addition to humans.^19^ Isatin was administered daily at a low (20 mg/kg/day; IL) or a high dose (100 mg/kg/day; IH) intraperitoneally during the last 14 days (days 15-28), similar to a previous study,^20^ and the PA-V group received saline solution. The health of the animals was monitored daily by food and water intake. Voluntary physical activities of the PA groups were recorded daily. On day 29, thiopental (100 mg/kg of body weight) was applied intraperitoneally for anesthesia for all rats, and the hearts were removed from the thorax.

Eight rats from the PA group were excluded from this study because they ran at a level below the inclusion criteria. During the isolated heart studies, 5 measurements in the control groups and 3 in the PA groups were excluded. This loss was due to technical difficulties in the experiment and the failure to appropriately measure critical parameters such as perfusion pressure, developed pressure (dP), heart rate, and heart rhythm. Thus, 16 rats in total were excluded, and a total of 68 of the 84 animals were included in this study.

In this study, female rats were investigated because they are more inclined to voluntary exercise on a running wheel than male rats.^21^ The estrous cycle in female rats lasts commonly 4.5 days;^22^ therefore, in the present study, the experimental process was continued for 3 consecutive cycles during the 14-day isatin application.

### Voluntary Physical Activity and Doses of Isatin

For voluntary PA, a stainless-steel running wheel was placed in the housing cage with a wheelbase of 7 cm height above the floor. The diameter of the wheel was 31.5 cm, and the width was 10 cm. The wheel’s circumference was 1081 cm, and the daily running distance was assessed by recording the frequency of rotation. The rats had free access to the running wheel. The animals in the control groups were housed individually in standard cages.

### Preparation of the Isatin Injection Solution

Isatin was obtained from Sigma Chemical Company (St Louis, Mo, USA). After weighing, the isatin powder was mixed with saline and shaken until the isatin was dissolved. The solution was prepared as 20 mg isatin in 5 mL saline for the low dose and 100 mg isatin in 5 mL saline for the high dose, and 1.25 mL was given to each rat. Thus, isatin was administered daily at a low dose (20-26 mg/kg; IL) or a high dose (95-144 mg/kg; IH) during the last 14 days (days 15-28). The isatin solution was administered using a 2 cc syringe with a 21G needle. The solution was prepared daily, kept at room temperature, and injected intraperitoneally.

### Isolated Heart Preparation and Measurements of Cyclic Guanosine Monophosphate in the Perfusion Solution

In subgroup 1, heparin was injected into the rats (500 IU/kg; Nevparin Vial Mustafa Nevzat, İstanbul, Turkiye) as an anticoagulant before anesthesia. Subsequently, the abdomen and thorax of the anesthetized rats were opened, and the heart was quickly removed and placed in a Petri dish containing cold Krebs-Henseleit solution for the isolated perfused heart model.^11^ Then, the aortic stump of the isolated heart was connected to the cannula of the Langendorff apparatus, and a latex balloon was inserted into the LV through the mitral valve. The heart was perfused with Krebs-Henseleit solution during a 15-minute equilibration period. Thereafter, left ventricular dP, the first derivative of LV dP during the systolic phase (dP/dt max), the first derivative of LV relaxation pressure during the diastolic phase (dP/dt min), and heart rates were recorded (Biopac MP36 System, Inc., Goleta, Calif, USA) during the 15 minutes. The Krebs-Henseleit solution used for the measurements was prepared daily and contained (in mmol/L) NaCl 118.3, NaHCO_3_ 25.0, KCl 4.7, KH_2_PO_4_ 1.2, MgSO4 1.2, CaCl_2_ 2.5, and glucose 11.1. The perfusate was equilibrated with 95% O_2_ and 5% CO_2_, and the hearts were perfused at a constant pressure of 65-70 mmHg, a temperature of 37°C and pH 7.4. The cGMP levels (BT-LAB, Shanghai, China) were assessed in perfusate samples, collected during the 15th minute of the experimental period, with the enzyme-linked immunosorbent assay (ELISA) method.

### Gene and Protein Analyses in Left Ventricle Tissue

In subgroup 2, the thorax was opened, and the hearts of the rats were removed quickly and weighed. Subsequently, the LV was separated and weighed. Thereafter, tissue samples were collected for further analysis.

For gene analysis, approximately 10-30 mg of ventricular tissue was homogenized using a homogenizer (RETSCH brand, MM 400 model, Haan, Germany). Total RNA was manually isolated from the homogenates using the Total RNA Miniprep Kit (EZ-10 Spin Column Total RNA Mini-preps Kit- BioBasic, Toronto, Canada). RNA concentrations and purities were then measured using a Nanodrop device, and the concentrations of the samples were equalized (Nanodrop ND-2000c- Thermo Fisher Scientific, Waltham, Mass, USA). The polymerase chain reaction (PCR) conditions were programmed as follows: Step 1 at 25°C for 10 minutes, Step 2 at 37°C for 120 minutes, and Step 3 at 85°C for 5 minutes using the cDNA Reverse Transcription Kit (OneScript® Plus cDNA Synthesis Kit, Abmgood Cat No: G236, Richmond, BC, Canada); cDNA synthesis was subsequently performed. The synthesized cDNAs were stored at −20°C for further analyses. The cDNAs for PDK-4, AKT, ERK1/2, and BNP genes were analyzed using appropriate primer-probe and mastermix (BlasTaq^TM^ 2XqPCRMaster Mix, Abmgood Cat No: G891, Richmond, BC, Canada) real-time PCR system as described previously.^23^ Primer sequences shown in Table 1 were used in real-time PCR. The results were calculated using the 2^−∆∆Ct^ method based on the Ct values of the peaks obtained during the amplification process. The glyceraldehyde-3-phosphate dehydrogenase (GAPDH) gene was used as a housekeeping gene to normalize expression values.

Protein kinase B, ERK1/2, and PDK4 protein levels in left ventricular tissue were analyzed by Western blotting.Cold Radio-Immune Precipitation Assay (RIPA) Lysis Buffer (Thermo Fisher Scientific, Waltham, Mass, USA) was used to homogenize the tissue samples. The protein content in the supernatant was determined by the Lowry method.^24^ Equal amounts of protein (30 µg) were subjected to a separation procedure on a 12% Sodium Dodecyl Sulfate Polyacrylamide Gel Electrophoresis (SDS-PAGE) and subsequently transferred to polyvinylidene fluoride (PVDF) membranes. The PVDF membranes were blocked with 5% skim milk for 1 hour. Thereafter, the membranes were incubated overnight at +4°C with PDK-4 Polyclonal Antibody (1:1000; Thermo Fisher Cat No: PA5-13776, Thermo Fisher Scientific, Waltham, Mass, USA), AKT Pan Monoclonal Antibody (1:1000; Thermo Fisher Cat No: MA514916, Thermo Fisher Scientific, Waltham, Mass, USA), ERK1/ERK2 Polyclonal Antibody (1:1000; Thermo Fisher Cat No: 61-7400, Thermo Fisher Scientific, Waltham, Mass, USA), and Beta Actin Monoclonal Antibody (1:1000; Thermo Fisher Cat No: PA1-183, Thermo Fisher Scientific, Waltham, Mass, USA). Then, the membranes were washed with tris buffered saline tween-20 (TBS-T) and incubated with the secondary antibody goat anti-Rabbit IgG (H+L) secondary antibody HRP (1:20000; Thermo Fisher Cat No: 31460, Thermo Fisher Scientific, Waltham, Mass, USA) for 1 hour. SuperSignal West Pico Chemiluminescent Substrate (Thermo Fisher Cat No: 34580, Thermo Fisher Scientific, Waltham, Mass, USA) was used to detect the protein bands. Image Lab software was used to measure the band intensity as described previously.^25^

### Statistical Analysis

Normality distribution of the numeric variables was evaluated by Shapiro-Wilk Test. Due to the non-normal distribution of the parameters, the Kruskal–Wallis test was used to compare statistically the voluntary activity of the physically active groups, cardiac hemodynamic parameters, cGMP, gene and protein analysis of all groups. When significance was detected, Dunn’s test with Bonferroni Correction was used to determine which group caused the difference. A total of 11 comparison pairs, including “C-V vs. C-IL,” “C-V vs. C-IH,” “C-V vs. PA-V,” “C-V vs. PA-IL,, “C-V vs. PA-IH,” “C-IL vs. C-IH,” “PA-V vs. PA-IL,” “PA-V vs. PA-IH,” “PA-IL vs. PA-IH,” “C-IL vs. PA-IL,” and “C-IH vs. PA-IH” were used. “PA-V vs. PA-IL,” “PA-V vs. PA-IH,” and “PA-IL vs. PA-IH” pairs were used to compare physical activities. The results were expressed as the mean ± SD and median (min-max). Statistical significance was accepted at *P *< .05. IBM SPSS 20 (IBM SPSS Corp.; Armonk, NY, USA) and GraphPad Prism (9.4.1) were used for data analysis and figural representation.

## Results

### Body Weight and Physical Activity

The initial body weights (IBWs) of rats did not significantly differ between C and daily exercise (PA) groups. The same holds for the body weights at the end of the experimental period (FBW). The small increase in body weight during the experimental period was also not statically different between the 6groups (*P *= .15). The mean values of daily running distance in the 3 PA groups did not statistically differ between the 3 PA groups: 4682, 5389, and 4801 m in group PA-V, PA-IL, and PA-IH, respectively (Table 2).

### Cardiac Hypertrophy Findings

Total heart weight/body weight ratio (HW/BW) did not differ between the 6 groups (Table 3). Left ventricular weight normalized on body weight did not differ between the 3 control groups; the same holds for the 3 PA groups. However, comparison of normalized left ventricular weight of the PA group receiving a high dose of isatin (PA-IH) with the corresponding control group receiving a high dose of isatin (C-IH) showed a significant increase (*P *= .02; Table 3). A post-hoc power analysis was used to calculate statistical power, and it was found as 80% with an alpha level of 5%, with a calculated effect size of 0.621 based on LV-W/BW, and sample size of the groups C-V (n = 7), C-IL (n = 7), C-IH (n = 7), PA-V (n = 6), PA-IL (n = 6), and PA-IH (n = 6).

### Hemodynamic Behavior of Isolated Hearts Perfused Ex Vivo and Cyclic Guanosine Monophosphate Levels in the Perfusate

**Figure 2.**
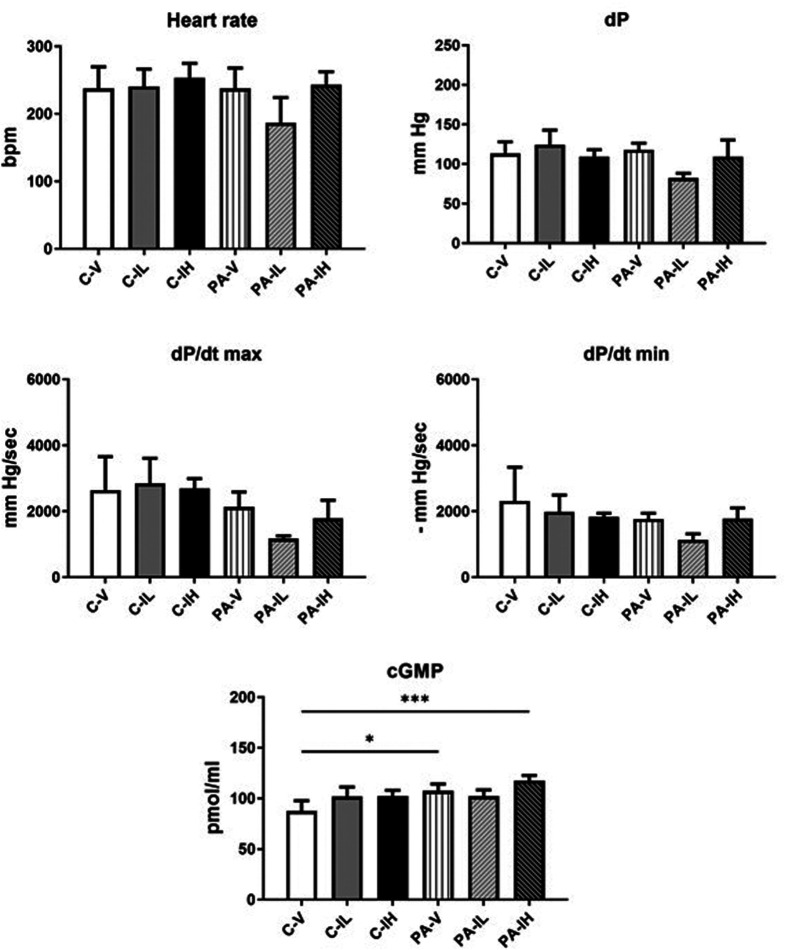
Effect of low- and high-dose isatin administration on heart rate and cardiac contractility of ex vivo perfused hearts, and cGMP concentration in the perfusate collected during the 15-minute perfusion; C-V, control-vehicle (n = 6); C-IL, control-isatin-low-dose (n = 5); C-IH, control-isatin-high-dose (n = 5); PA-V, physical activity-vehicle (n = 6); PA-IL, physical activity-isatin-low-dose (n = 3); PA-IH, physical activity-isatin-high-dose (n = 4) groups. Values are expressed as mean ± SD. ^*^P < .05, ^***^P < .001.

Heart rate of isolated hearts perfused ex vivo during the 15-minute measurement period did not significantly differ between group C-V (n = 6), C-IL (n = 5), C-IH (n = 5), PA-V (n = 6), PA-IL (n = 3), and PA-IH (n = 4). The same holds for left ventricular dP, and the rate of contraction (dP/dt max) and relaxation (dP/dt min) (Figure 2).

Perfusate cGMP levels did not differ between the 3 C groups. The same holds for comparing the 3 PA groups. However, when comparing the PA-V and PA-IH groups with the non-running control group injected with saline, group C-V, a significant increase in cGMP release from the ex vivo perfused hearts of the 2 PA groups was observed.

### Gene and Protein Expressions in the Left Ventricle

**Figure 3.**
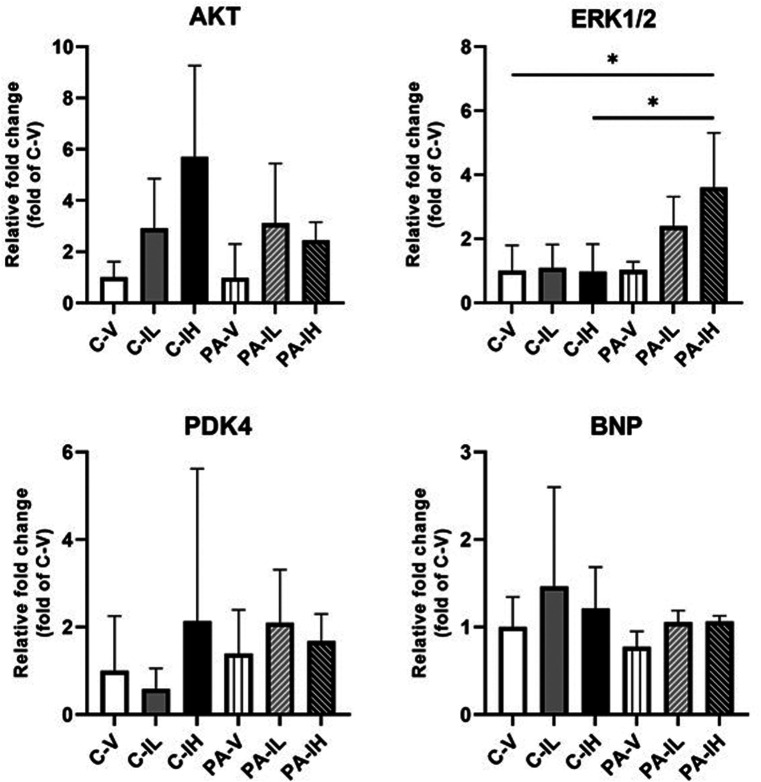
Effect of low- and high-dose isatin administration on the protein kinase B (AKT), extracellular-regulated kinase 1/2 (ERK1/2), pyruvate dehydrogenase kinase-4 (PDK-4), and B-type natriuretic peptide (BNP) gene expressions in left ventricle tissue in C-V, control-vehicle (n = 7); C-IL, control-isatin-low-dose (n = 7); C-IH, control-isatin-high-dose (n = 7); PA-V, physical activity-vehicle (n = 6); PA-IL, physical activity-isatin-low-dose (n = 6); and PA-IH, physical activity-isatin-high-dose (n = 6) groups. Values are expressed as mean ± SD. ^*^P < .05.

Protein kinase B gene expression levels in the left ventricular tissue were found to be similar in the C-V (n = 7), C-IL (n = 7), C-IH (n = 7), PA-V (n = 6), PA-IL (n = 6), and PA-IH (n = 6) groups (Figure 3). The expression level of the *ERK1/2* gene was not significantly different between the 3 control C groups. The same holds for the 3 running PA groups of rats. However, the *ERK1/2* gene expression level was significantly higher in the running PA group receiving a daily high dose of isatin (PA-IH group) when compared with the corresponding non-running group C-IH and the control group C-V. No significant differences in the expression level of *PDK-4* and *BNP* genes were present between the 6 experimental groups (Figure 3).

**Figure 4.**
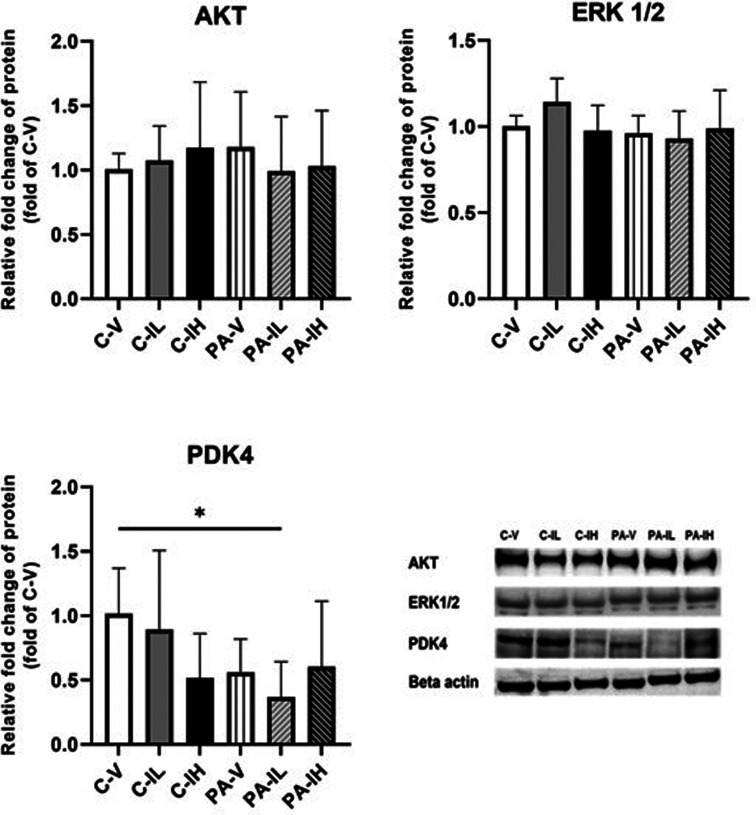
Effect of low and high dose isatin administration on the protein kinase B (AKT), extracellular-regulated kinase 1/2 (ERK1/2), pyruvate dehydrogenase kinase-4 (PDK-4) protein expressions in left ventricle tissue in the C-V, control-vehicle (n = 7); C-IL, control-isatin-low-dose (n = 7) and C-IH, control-isatin-high-dose (n = 7); PA-V, physical activity-vehicle (n = 6); PA-IL, physical activity-isatin-low-dose (n = 6); PA-IH, physical activity-isatin-high-dose (n = 6) groups. Values are expressed as mean ± SD. ^*^P< .05.

With respect to protein levels, no differences were observed between the 6 experimental groups in AKT and ERK1/2. PDK-4 protein expression was similar in the 3 non-running C groups. The same holds for the 3 running PA groups. However, when the protein level of PDK-4 is compared with that of the C-V group, a significantly lower value in the PA-IL group was observed (Figure 4).

## Discussion

The results of the present study provided evidence that prolonged treatment with isatin in female rats, when allowed voluntary running in a running wheel, is very well tolerated. In addition, the lack of a negative effect of long-term isatin treatment on daily PA was corroborated by findings on cardiac performance. Comparison of the performance of hearts obtained from rats with no access to a running wheel and from rats allowed to perform enhanced daily exercise did not reveal any negative effect of isatin treatment on heart rate, dP, and contraction and relaxation rate when perfused ex vivo. However, the present study showed that isatin may cause a dose-dependent effect on cGMP, ERK1/2 gene expression, and PDK-4 levels in the LVs of female rats performing voluntary PA.

Several behavioral studies have revealed an acute, negative effect of isatin on locomotor activity and open-field mobility of experimental animals.^3^^-^^5^ However, no information is present on the long-term effect of isatin on skeletal muscle activity. The therapeutic role of isatin has been investigated in previous studies using low doses (5-20 mg/kg) or high doses (>50 mg/kg) isatin.^2^^,^^11^^,^^20^ In this study, the running distance was over 4.6 km per day in rats not treated with isatin. The mean daily running distance levels of physically active groups treated with low or high doses of isatin were over 6.1 and 4.8 km, respectively, which is a statistically non-significant difference compared to the running distance of saline-treated animals.

Until now, conflicting findings have been reported on the relationship between exercise and BNP release in the heart. BNP increases due to physiological overload during physical exercise.^17^ However, exercise could prevent the elevation of BNP in cardiac tissue.^26^ Tourki and coworkers^27^ reported that isatin probably had an inhibitory effect on cardiac contractility and that this effect was due to the suppression of the potent cardioprotective effect of BNP. This study, showing a lack of alteration of cardiac BNP gene expression in rats, is not in line with a potential role of BNP in alterations of cardiac performance, if any.

In this study, cGMP levels were higher in the PA-V and PA-IH groups, but this increase seemed to be somewhat suppressed in the PA-IL group. cyclic guanosine monophosphate provides PKG-mediated phosphorylation and has an inhibitory role in cardiac hypertrophy.^28^ The findings of this study indicate that the possible cardiac hypertrophic effects of cGMP show dose-dependent changes. To date, the knowledge regarding low- and high-dose isatin on the cell signaling pathways varies depending on concentration.^29^ According to the authors’ findings from physically active rats that were not treated with isatin or treated with high doses of isatin, cGMP levels may have increased due to PA. This increase in cGMP may result from adaptation to exercise due to nitric oxide production.^30^ However, exposure to isatin in low doses may suppress the cGMP level in physically active rat hearts.

Studies conducted so far show an improvement in cardiac function in the case of a decrease in PDK-4 levels.^16^ Pyruvate dehydrogenase kinase-4 impairs ventricular function by inhibiting pyruvate dehydrogenase.^15^ Despite the deleterious cardiac effect of a decrease in PDK-4, this enzyme increases in muscle during PA, depending on the intensity of exercise.^31^ In the present study, cardiac PDK-4 gene and protein levels did not change in the PA-V group compared to C-V. Similar findings were obtained when these rats received low or high doses of isatin, justifying the conclusion that neither voluntary exercise nor isatin exerts a negative or positive effect on cardiac PDK-4 and, hence, the contribution of glucose to overall energy conversion. However, a significant decrease in PDK-4 protein levels was observed in the PA-IL group compared to the C-V group. This finding suggests that PA and low-dose isatin administration may have a combined metabolic effect. Theoretically, a decline in PDK-4 enzyme activity might have a positive impact by activating glucose metabolism; it is noteworthy that in the present study this effect is not associated with a marked change in ventricular function.

Isatin treatment reduces blood pressure,^9^ most likely due to its vasodilator effect on smooth muscle cells.^10^ Cardio-depressive effects have been reported in isolated frog hearts.^9^ In this study, hemodynamic similarity and consistent AKT and ERK protein levels in groups suggest that the heart can tolerate long-term isatin administration under conditions with or without voluntary wheel running.

The study strongly suggests a combined effect of physical exercise and a high dose of isatin on left ventricular growth. This hypertrophic effect was absent in rats receiving a lower dose of isatin. Maillet and coworkers^32^ reported that physical exercise-induced cardiac hypertrophy combined with volume overload was caused by AKT activation. However, the authors’ study does not indicate the involvement of AKT activation in the cardiac hypertrophic response in the physically active rats receiving a high dose of isatin.

In terms of isatin administration of ERK, Cane and colleagues^33^ observed that isatin strongly inhibited ERK1/2 activity. In addition to a physiological adaptive hypertrophic response to physical exercise, ERK1/2 activation in cardiac tissue is associated with a maladaptive hypertrophic change triggered by chronic diseases or prolonged stress.^14^ Since ERK1/2 protein rather than ERK1/2 mRNA is supposed to exert a biological action, the authors are inclined to conclude that ERK1/2 does not play a significant role in the effect of isatin in combination with exercise on ventricular growth.

This study investigated the effect of isatin on healthy animals. However, isatin has a therapeutic potential in Parkinson’s disease rat model^18^ and high-dose isatin injection positively affected muscle motor activity in the rat Parkinson model.^12^ In this study, isatin administered to healthy rats at the high dose caused a non-significant difference with respect to running distance when compared to saline-treated animals. Future studies are needed to examine how isatin treatment affects PA levels in humans. The present study also reveals isatin-induced molecular changes in the cardiac hypertrophic signaling pathways in physically active rats. Therefore, it would be appropriate to take PA levels into account in future studies examining the cardiac effects of isatin in humans.

The findings of this study may be limited in addressing the role of isatin on daily PA and cardiac performance in both genders. In addition, since each female rat underwent approximately 3 consecutive estrous cycles during the treatment period, interindividual differences of the estrus cycle on the study outcome were considered to be minimal.^34^ Moreover, literature data indicate that physiologic concentrations of estradiol generated during the estrous cycle do not influence cardiovascular parameters.^35^ It is of note that the willingness to perform voluntary physical exercise seen in rats may not be evident in humans. In studies on human subjects, the inclusion of both sexes is, therefore, advisable. In addition, the findings of this study may have other limitations in addressing the low sample size in the hemodynamic measurements.

In summary, the findings of the present study demonstrated that long-term treatment with isatin is well tolerated without impairment of daily PA and does not change cardiac hemodynamic performance in female rats. However, in rats performing voluntary PA, differences may be observed in ventricle growth and molecular and metabolic processes depending on the dose of isatin. In physically active female rats, high doses of isatin may affect ERK, a molecule associated with exercise-induced ventricular growth, whereas low doses of isatin appear to have more pronounced effects on suppressing cGMP and reducing PDK-4 levels in the LV, which highlights the dose-dependent differences in future studies for isatin.

## Figures and Tables

**Table 1.** The Primer Sequences Were Utilized in Real-time Polymerase Chain Reaction

Gene	Sequences	GeneBank
*PDK4*	F: 5’- GAACACCCCTTCCGTCCAGCT-3’ R: 5’- TGTGCCATCGTAGGGACCACA -3’	XM_032906966.1
*ERK1/2*	F: 5’- CTCGGA TTCCGCCATGAGAA -3’ R: 5’- GGTCGCAGGTGGTGTTGATA -3’	X65198.1
*AKT*	F: 5’- TCACCTCTGAGACCGACACC -3’ R: 5’- CCGTTCACTGTCCACACACTC -3’	XM_032908539.1
*BNP*	F: 5’- GATGCAGAAGCTGCTGGAGCT -3’ R: 5’- ATCCGGAAGGCGCTGTCTTG -3’	XM_032887581.1
*GAPDH*	F:5’- GGCACAGTCAAGGCTGAGAATG -3’, R:5’- ATGGTGGTGAAGACGCCAGTA -3’;	XM_032902285.1

AKT, protein kinase B; BNP, B-type natriuretic peptide; ERK1/2, extracellular signal-regulated kinase1/2; GAPDH, glyceraldehyde-3-phosphate dehydrogenase; PDK4, pyruvate dehydrogenase kinase-4.

**Table 2.** Body Weight and Physical Activity in Running Wheel

Group	C-V (n = 13)	C-IL (n = 12)	C-IH (n = 12)	PA-V (n = 12)	PA-IL (n = 9)	PA-IH (n = 10)	*P*
IBW (g)	226 ± 25 233 (182/259)	209 ± 13 205 (193/234)	217 ± 10 218 (200/230)	224 ± 11 225 (208/244)	218 ± 14 217 (201/238)	212 ± 23 205 (174/250)	.085
FBW (g)	240 ± 27 242 (196/270)	215 ± 13^*^ 212 (200/244)	223 ± 9 223 (211/239)	229 ± 14 229 (212/256)	223 ± 13 218 (206/245)	223 ± 23 218 (189/263)	.046
Δ Change body weight (g)	14.2 ± 12.2 10.0 (−3.0/47.0)	5.8 ± 4.9 6.0 (−4.0/14.0)	6.4 ± 6.2 5.0 (−1.0/20.0)	4.9 ± 9.4 7.0 (−12.0/15.0)	5.3 ± 11.5 5.0 (−20.0/15.0)	10.8 ± 7.2 11.5 (0.0/19.0)	.131
Daily PA (m)				4682 ± 3131 3415 (831/9735)	5389 ± 3933 5351 (1448/14009)	4801 ± 2944 3549 (490/8327)	.978

Values are expressed as mean ± SD and median (min-max). C-V, control-vehicle; C-IL, control-isatin-low-dose; C-IH, control-isatin-high-dose; FBW, final body weight and daily; IBW, initial body weight; PA, daily physical activity refers to distance (m) run in the running wheel (PA-groups only); PA-V, physical activity-vehicle; PA-IL, physical activity-isatin-low-dose; PA-IH, physical activity-isatin-high-dose groups. *P < .05 compared with C-V group.

**Table 3.** Heart and Left Ventricular Weight Normalized on Body Weight

Group	C-V (n = 7)	C-IL (n = 7)	C-IH (n = 7)	PA-V (n = 6)	PA-IL (n = 6)	PA-IH (n = 6)	*P*
HW/BW (mg/g)	3.4 ± 0.3 3.3 (3.0/4.0)	3.2 ± 0.2 3.2 (3.0/3.7)	3.1 ± 0.3 3.2 (2.6/3.5)	3.2 ± 0.1 3.2 (3.0/3.3)	3.4 ± 0.3 3.3 (3.2/3.9)	3.4 ± 0.3 3.4 (3.0/3.7)	.143
LVW/BW (mg/g)	2.2 ± 0.1 2.2 (2.0/2.5)	2.2 ± 0.2 2.2 (1.9/2.6)	2.1 ± 0.2 2.1(1.7/2.5)	2.2 ± 0.1 2.2 (2.0/2.4)	2.3 ± 0.1 2.2 (2.2/2.4)	2.4 ± 0.2^#^ 2.4 (2.3/2.7)	.022

Values are expressed as mean ± SD and median (min-max). C-V, control-vehicle; C-IL, control-isatin-low-dose; C-IH, control-isatin-high-dose; HW/BW, heart weight/body weight; LVW/BW, left ventricle weight/body weight; PA-V, physical activity-vehicle; PA-IL, physical activity-isatin-low-dose; PA-IH, physical activity-isatin-high-dose groups. ^#^*P* < .05 compared with C-IH group.

## Data Availability

The data that support the findings of this study are available on request from the corresponding author.
